# Active Compounds in Fruits and Inflammation in the Body

**DOI:** 10.3390/nu14122496

**Published:** 2022-06-16

**Authors:** Magdalena Majdan, Barbara Bobrowska-Korczak

**Affiliations:** Department of Bromatology, Faculty of Pharmacy, Medical University of Warsaw, Banacha 1, 02-097 Warsaw, Poland; mmajdan@wum.edu.pl

**Keywords:** fruits, inflammation, antioxidant, phytochemical compounds

## Abstract

Inflammation plays an important role in the pathogenesis of many diseases, including cardiovascular diseases, atherosclerosis, diabetes, asthma, and cancer. An appropriate diet and the active compounds contained in it can affect various stages of the inflammatory process and significantly affect the course of inflammatory diseases. Recent reports indicate that polyphenolic acids, vitamins, minerals, and other components of fruits may exhibit activity stimulating an anti-inflammatory response, which may be of importance in maintaining health and reducing the risk of disease. The article presents the latest data on the chemical composition of fruits and the health benefits arising from their anti-inflammatory and antioxidant effects. The chemical composition of fruits determines their anti-inflammatory and antioxidant properties, but the mechanisms of action are not fully understood.

## 1. Introduction

Epidemiological, toxicological, and nutritional studies indicate a link between fruit consumption and the reduced incidence of chronic diseases, such as coronary disease, cancers, diabetes, and neurodegenerative diseases. The growing interest in fruits as dietary components mainly stems from their antioxidant and anti-inflammatory potential. The last few years have seen increased interest in fruits as raw plant materials with anti-inflammatory properties. Research has focused on explaining the mechanisms of anti-inflammatory and antioxidant activity. The health effects of the active substances present in fruit also include the effective detoxification of the body, increased immune system activity, the equalization of blood pressure, and anti-aggregation activity, as well as antibacterial and antiviral properties.

The consumption of red meat and highly processed food has been shown to lead to the development of pro-inflammatory processes, whereas a healthy and active lifestyle and a diet rich in fruit and vegetables are correlated with the prevention of inflammatory diseases and support their treatment [1,2]. Bioactive substances in fruits and vegetables, including flavonoids, carotenoids, vitamins, minerals, and dietary fiber, can act independently or synergistically to provide high nutritional value and health benefits for consumers [3,4].

## 2. Targets of the Anti-Inflammatory Action

**Figure 1:**
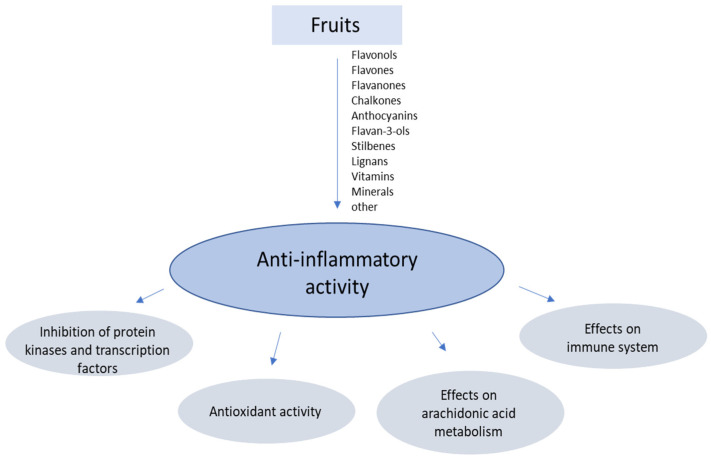
Selected targets of the anti-inflammatory activity of bioactive substances in fruits.

Phytochemical compounds, vitamins, and minerals present in fruit exhibit anti-inflammatory properties through a variety of mechanisms, including the inhibition of regulatory enzymes, free radical scavenging, and the regulation of arachidonic acid metabolism, gene expression, and immune cell activity (Figure 1).

### 2.1. Inhibition of Protein Kinases and Transcription Factors

Protein kinases are involved in signal transduction during the activation of cells during inflammation. Certain chemical compounds present in fruit can affect the activity of protein kinases by inhibiting transcription factors, such as NF-κB [5]. This is one of the most important transcription factors regulating the expression of cytokines, chemokines, immune receptors, and cellular adhesion molecules [6]. NF-κB is present in the cytoplasm of cells in an inactive form bound to regulatory proteins known as κB inhibitors (IκB), including IκBα, IκBβ and IκBϵ. The activation of NF-κB requires its release from the NF-κB–IκB complex, which is mediated by inhibitor of the nuclear factor kappa-B kinase (IKK). The IKK complex consists of three subunits, of which IKK beta (β) may play the most important role in chronic inflammation. The activation of NF-κB further leads to the transcription of genes coding for molecules, including cytokines, which are usually involved in the inflammation process. Similarly, AP-1, a transcription factor involved in inflammation, is stimulated by stress factors, including infections and cytokines. Other important mediators of inflammation include pattern recognition receptors, such as toll-like receptors (TLR), and kinases, such as the mitogen-activated protein kinase (MAPK) and the stress-activated kinase JNK [7]. CD4+ T lymphocytes can differentiate into subpopulations performing helper functions in the body (Th2). Some plant flavonoids are believed to be competitive inhibitors of PDE4 (cyclic nucleotide phosphodiesterase 4) and to modulate inflammation in the liver by altering cAMP (cyclic adenosine monophosphate) levels [8,9].

### 2.2. Antioxidant Activity

Clinical symptoms of inflammation are induced by fatty acid derivatives (eicosanoids), the platelet-activating factor (PAF), large proteins, such as IL-1, small peptides (bradykinin), and amines (histamine) released from damaged and migrating cells as a consequence of increased vascular permeability. Nitrogen monoxide can perform a regulatory function at every stage of the development of inflammation, including the regulation of the pro-inflammatory properties of the endothelium and the early stages of leukocyte migration to the site of inflammation. The activity of this isoenzyme depends on the concentration of pro-inflammatory cytokines (TNF-α, IFN-γ and IL-1). Released NO• together with other reactive oxygen species plays an important role in the development of inflammatory reactions and the regulation of leukocyte adhesion to endothelial cells, which is the initial stage of inflammation, dependent on the expression of numerous adhesion molecules on the surface of both types of cells. The expression of adhesion molecules is stimulated by various cytokines, bacterial LPS, and reactive oxygen species (ROS). Activated monocytes and neutrophils release myeloperoxidase, which produces hypochlorous acid (I) (HClO) involved in damage to amino acids, proteins, fats, and nucleic acids. Another important mechanism is the formation of chloramines and their later conversions. The reactions of HClO with proteins generating free radicals are also significant, which contribute to adduct formation and protein fragmentation [10]. In normal conditions, living organisms are protected against the effects of free radicals by the activity of enzymes, such as superoxide dismutase and glutathione peroxidase and the presence of natural antioxidants—ascorbic acid, polyphenols, tocopherols, and carotenoids. When cells are exposed to an excessive level of oxidants, including free radicals, natural defense mechanisms fail. This results in free radical damage to cellular structures involving destruction within the cell membranes—lipid peroxidation, deactivation of structural and enzymatic proteins, and damage to genetic material. Fruits are believed to be a valuable source of a variety of antioxidants, e.g., vitamins C and E, carotenoids, and polyphenols—anthocyanins, flavonoids, phenolic acids, and tannins. Studies have shown that antioxidants neutralize free radicals, which are reactive oxygen species, peroxide radicals, and singlet oxygen. In addition, they protect against collagen degradation and inhibit the activity of xanthine oxidase. Antioxidants also reduce capillary permeability and exert a strong anti-inflammatory effect [11].

### 2.3. Effects on Arachidonic Acid Metabolism

Prostaglandins, thromboxanes, and leukotrienes are mediators inducing inflammation. Arachidonic acid (AA) is released from membrane phospholipids as a result of the activity of phospholipase A2 (PLA2). Released AA is then converted to prostaglandins and thromboxanes in a series of enzymatic reactions catalyzed by cyclooxygenases (COX). Similarly, the activity of 5-lipoxygenase (5-LOX) catalyzes the conversion of arachidonic acid to leukotrienes [12].

### 2.4. Effects on the Immune System

Some substances in fruits promote the activation of cells, signal transduction, cytokine production, or secretory processes in certain immune cells. Flavonoids inhibit the maturation of dendritic cells (DC) in the mesenteric lymph nodes and human MDDCs (monocyte-derived dendritic cells), and subsequently the function of CD4+ T-cells mediated by DC in vitro [13,14]. Phenolic compounds can have an inhibitory effect on monocyte adhesion and/or reduce the activation and proliferative response of certain immune cells involved in chronic inflammation [15]. Certain flavonoids reduce the release of histamine or prostaglandin from mast cells or inhibit production of pro-inflammatory cytokines or chemokines in mast cells, neutrophils, and other immune cells [16]. 

## 3. Inflammation and Tumor Induction

Carcinogenesis in humans is characterized by the accumulation of numerous mutations in genes regulating cellular homeostasis, primarily oncogenes, suppressor genes, and genes regulating apoptosis. Mutations are caused by xenobiotics and endogenous DNA damage, generated throughout a person’s lifetime and affecting every stage of carcinogenesis. The main factor causing this endogenous damage not only to DNA but also to lipids and proteins is reactive oxygen and nitrogen species generated in inflammatory states by neutrophils and macrophages stimulated by this process [17]. The source of endogenous ROS in physiological conditions is the respiratory chain. Endogenous ROS and RNS react directly with DNA, modifying its structure and functions [17,18]. Inflammation and mutations induced by exogenous (environmental) factors are therefore linked. Cells stimulated during inflammation can intensify the carcinogenesis process, sometimes as the result of other mechanisms, such as the production of cytokines or other proteins that increase the proliferation and invasiveness of preneoplastic cells, as well as the formation of new blood vessels in the angiogenesis process [19].

Inflammation plays a decisive role at various stages of tumor development, including initiation, promotion, malignant transformation, and invasion during metastasis. Its presence affects the immune system and the response to therapy. Environmental factors affecting cancer development, such as chronic infections, polluted air, or obesity, are associated with the occurrence of chronic inflammation [20].

Some studies suggest that as many as 50% of tumors may be associated with inflammation, which has given rise to the concept of ‘cancer-related inflammation’. Anti-inflammatory treatment has been shown to contribute to the prevention and treatment of cancer, confirming their correlation. Immune cells have been shown to be involved in various stages of tumor formation, which has enabled the development of new cancer treatment strategies [21,22].

## 4. Mechanism of the Anti-Inflammatory Action of Phenolic Compounds in Fruits

### 4.1. Flavonoids

An increasing number of scientific reports indicate that polyphenolic compounds, such as flavonoids, can inhibit regulatory enzymes or transcription factors playing important roles in the inflammatory process. Flavonoids are also known as compounds with antioxidant properties [23].

Flavonoids are polyphenolic compounds that are widespread in the plant kingdom. They are abundant in fruits such as blackberries, blueberries, raspberries, black currants, strawberries, grapes, cranberries, apples, and sour cherries [24]. They protect plants against damage caused by pathogens, wounds, or excessive UV radiation. Flavonoids are usually present as yellow pigments dissolved in cell sap in flowers and leaves and as red and blue pigments in fruits. Flavonoids are the predominant phenolic compounds in the diet. They are most often found in bound form as glycosides or esters, and less often as free compounds (Table 1) [25].

Studies have shown that the number and position of hydroxyl groups is of key importance for the chemical structure of flavonoids and their biological activity. Flavonoids present in fruits are bound to sugars. The skeleton of flavonoids consists of diphenylpropane, two benzene rings connected by a three-carbon chain, which forms a closed heterocyclic pyran ring with a benzene ring. Their subclasses are flavonols (apples and berries), flavanones flavones (citrus fruits), flavonols, isoflavones, flavan-3-ols, and anthocyanins (blueberries, strawberries, cherries, and grapes) [25,26].

The degree of glycosylation directly influences the antioxidant capacity of flavonoids. In vitro research has shown that aglycone forms of myricetin and quercetin are more active than glycoside forms. The antioxidant activity of flavonols is determined by the carbohydrate moiety, and the differences in activity can be explained by the varied oxidizability of glycosides containing a monosaccharide or disaccharide at carbon 3 [27].

#### 4.1.1. Flavonols

The set of flavonoids in many fruits includes flavonol derivatives, quercetin and kaempferol, and their glycosides: rutoside, glucoside, galactoside, and glucuronide. Flavonols, particularly quercetin derivatives, have strong antioxidant properties, which together with other active compounds are responsible for the therapeutic effect of fruits. At the same time, quercetin exhibits properties inhibiting cyclooxygenase and lipoxygenase, enzymes involved in arachidonic acid conversions, thereby reducing the production of prostaglandins and leukotrienes [28,29]. Apples, cranberries, chokeberries, blueberries, grapes, raspberries, sour cherries, and black currants are the richest fruit sources of quercetin [30,31,32]. Quercetin is described as one of the strongest antioxidants protecting against oxidative stress induced by amyloid deposits [33].

Black currants contain flavonols, such as myricetin, quercetin, and, in smaller quantities, kaempferol [34]. Consumption of black currants has been shown to increase the quercetin concentration in human serum. Similar results have been obtained for consumption of European blueberries and lingonberries [35].

Rutin (quercetin 3-O-rhamnoglucoside) is a flavonol glycoside, a component of quercetin bound to rutinose by a glycoside bond. It is present in apricots, sour cherries, grapes, grapefruit, plums, and oranges [36,37]. Rutin exhibits antioxidant activity. It can donate electrons to free radicals, converting them to more stable and less reactive forms. Rutin can also prevent oxidative stress by inhibiting enzymes responsible for ROS production, which can play a role in the treatment of diseases associated with oxidative stress, e.g., neurodegenerative diseases [38]. The anti-inflammatory activity of rutin, together with quercetin and hesperidin, has been tested in rats using a model of acute and chronic inflammation. The daily intraperitoneal administration of flavonoids inhibited both acute and chronic stages of induced inflammation. Rutin was found to be most active in the chronic stage [39]. The anti-inflammatory activity of rutin may be due to its modulation of expression of the ASC complex (apoptosis-associated speck-like protein), which mediates inflammation [40]. An experimental colitis model showed that rutin has an anti-inflammatory effect on the intestines. Reduced secretion of pro-inflammatory cytokines (IFNγ and TNF-α) by the mesenteric lymph nodes was observed. The RT-qPCR method was used to show that the use of rutin resulted in an 80% decrease in the expression of pro-inflammatory genes in the cells of the large intestine, including IFNγ, TNF-α, and IL-1β. The reduced activation of splenic CD4+ cells (the phosphorylation of STAT4 and the expression of IFNγ) and lower cytokine concentrations in the plasma were noted in mice receiving flavonoids. This effect was also seen in the lymphocytes of the mucosa, which was linked to a reduced phosphorylation of STAT4 [41]. Research has shown that oral administration of rutin (100 mg/kg BW) in a study conducted using the plethysmometric method resulted in a reduction in a λ-carrageenan-induced oedema of rat paws. Rutin significantly inhibited the chemotaxis of neutrophils stimulated with fMLP (N-formylmethionyl-leucyl-phenylalanine). In addition, elastase exocytosis induced by fMLP and cytochalasin B was partially inhibited by rutin [42]. The subject of another study on the anti-inflammatory activity of rutin was human umbilical vein endothelial cells (HUVECs). HMGB1 (high mobility group box 1) protein acts as a late mediator of severe vascular inflammation. Pro-inflammatory responses in HUVECs were induced by HMGB1 and the associated signaling pathways. Rutin inhibits the release of HMGB1, deactivates HMGB1-dependent processes in human endothelial cells, and reduces hyperpermeability and leukocyte migration mediated by HMGB1 in mice. Rutin was shown to inhibit the production of TNF-α and IL-6 and the activation of NF-κB and extracellular signal-regulated kinases ½ by HMGB1 [43]. The latest in vitro studies show that rutin inhibits the secretion of NO and TNF-α and the activity of MPO (myeloperoxidase) in a model of activated human neutrophils [44]. Interesting reports on the anti-inflammatory activity of flavonoids include results obtained in an in vitro model indicating the varied effects of different flavonoids on TNF-α production in LPS-stimulated J774.1 cells. Flavonoids, such as flavones, flavonols, and chalcones, were observed to inhibit production of TNF-α. Flavanone (naringenin) and anthocyanidins (pelargonin and cyanidin) showed moderate activity inhibiting the secretion of tumor necrosis factor. The double bond between carbons 2 and 3 and the ketone group in position 4 in the structure of flavonoids were shown to be responsible for the strong inhibitory effect on secretion of TNF-α [45].

#### 4.1.2. Flavones

One of the best-known compounds in terms of anti-inflammatory effects is apigenin (4′,5,7-trihydroxylaflavon). This compound, which is one of the flavones, is present in fruits, such as apples, sour cherries, grapes, oranges, and lemons. Apigenin inhibits synthesis of prostaglandin E2 (PGE2) and the activity of cyclooxygenase (COX-2). Evidence of its anti-inflammatory effects was presented by Liang et al. [46]. The authors demonstrated the dose-dependent suppression of nitric oxide (II) and prostaglandins through the suppression of the enzymes nitric oxide synthase (iNOS) and COX-2 in LPS-activated RAW 264.7 macrophages. Apigenin inhibits the activity of NF-kB-dependent pathways. It has been shown to inhibit LPS- or INFλ-induced activity of the IkB kinase. Research suggests that apigenin and related flavonoids have a role in preventing carcinogenesis and inflammation. Th17 lymphocytes mediate the appearance of inflammation in diseases, such as Crohn’s disease and psoriasis, as well as cancers associated with the overexpression of COX-2 (breast and colon cancer). Apigenin also causes the apoptosis of hyperactive antigen-presenting cells and T and B cells in lupus, most likely by inhibiting the expression of anti-apoptotic molecules regulated by NF-κB, especially COX-2 and the apoptosis regulator c-FLIP, which are constantly overexpressed by immune cells in lupus [47]. Another study showed that apigenin inhibits the LPS-initiated production of pro-inflammatory cytokines, such as IL-6, IL-1β, and TNF-α, through the modulation of many intracellular signaling pathways in macrophages in a model of THP-1-induced human macrophages and murine J774A.1 macrophages [48].

#### 4.1.3. Flavanones

Flavanones are abundant in citrus fruits. Naringenin (5,7,4′-trihydroxyflavon) is present in grapefruits, oranges, and mandarins, most often in the form of rutinosides with sugar fragments, glucose, and rhamnose. In vitro studies have shown that these compounds exhibit antioxidant and anti-inflammatory activity [49]. Besides naringenin and its derivatives, citrus fruits have also been shown to contain rutin (a flavonol glycoside) and tangeretin (a methylated flavone) [24]. Narirutin and naringin are dominant in grapefruit; hesperidin and narirutin in oranges; and eriocitrin in lemons. A recent review of the biochemical and pharmacological activity of citrus flavanones emphasized correlations between their structure and their function and ability to modulate signaling cascades both in vitro and in vivo [26]. In hesperidin, a lipophilic chain attached to a 7-hydroxyl group was shown to increase the anti-inflammatory effect [50]. Another study showed that both hesperidin and diosmin inhibited the synthesis and biological activity of various pro-inflammatory mediators, mainly arachidonic acid derivatives, prostaglandins E2 and F2, and thromboxane A2 [51].

Apples, sour cherries, grapes, lemons, and pomegranates contain luteolin (3′,4′,5,7-tetrahydroxyflavon) and its derivatives. In addition to its anti-inflammatory effects, antibacterial, cardioprotective and anti-tumor effects have been described as well [52,53,54]. Luteolin has been shown to protect mice against acute pancreatitis, inducing anti-inflammatory and antioxidant activity mediated by HO-1, in combination with the suppression of the activation of the NF-κB pathway [55]. Another study demonstrated that luteolin reduces the concentrations of TNF-1α, IL-1β, and IL-6 and may protect against changes in cognitive functions and synaptic plasticity induced by chronic cerebral hypoperfusion [56]. Luteolin, together with apigenin, inhibits the production of Th2 cytokines, such as IL-4 and IL-5 or IL-13, by activated human basophils [57].

#### 4.1.4. Chalcones

Chalcones are lipophilic compounds with a yellow color. The characteristic feature of the chalcone molecule is an open heterocyclic ring whose closure transforms the chalcone into a flavanone configuration. Chalcones are unstable compounds [58]. Dihydropochalcones (such as phloridzin) are present in apples and apple products (juice, cider, and pomace) [59]. The naringenin chalcone exhibits anti-inflammatory properties by inhibiting monocyte chemotactic protein MCP-1, TNF-α, and NO in LPS-stimulated RAW 264 macrophages and reduces the expression of iNOS [60]. The hesperidin methyl chalcone inhibits the carrageenan-induced production of cytokines (TNF-α, IL-1β, IL-6, and IL-10) and influences the level of oxidative stress and activation of NF-kB [61].

#### 4.1.5. Anthocyanins

Anthocyanins are water-soluble pigments included among polyphenols, commonly present in plants and responsible for the colors red, purple, and blue. Numerous reports based on vitro cultures, animal models, and clinical trials indicate the health benefits of plant extracts rich in anthocyanins. Anthocyanins are highly unstable compounds that are rapidly degraded. The stability of the compound depends on numerous factors, including pH, temperature, light, the chemical structure of the compound, its concentration, and the presence of enzymes, flavonoids, proteins, and metal ions [62].

Anthocyanins differ in the number of hydroxyl groups in the molecule; their level of methylation; the site, form, and number of attached sugar molecules; and the number and form of aromatic and aliphatic acids attached to the sugars. In berry fruits, anthocyanins are present in various (mono-, di-, or tri-) glycoside forms, where the glycoside residues are usually substituted at C3, or rarely at C5 or C7. The dominant sugars are sophorose, sambubiose, rutinose, arabinose, rhamnose, galactose, and glucose (Table 2) [63]. Many anthocyanins are acylated by aliphatic or aromatic acids, such as coumaric, caffeic, ferulic, p-hydroxybenzoic, sinapic, malonic, acetic, succinic, oxalic, and malic acids [64].

Xie et al. [65] conducted an in vivo study on mice receiving supplements of blueberry extract. The phytochemical components of the extract were shown to effectively inhibit the production of the inflammatory cytokines TNF-α and IL-6 in murine macrophages by influencing the signaling pathways involved in their production (the inhibition of the phosphorylation of IκB, NF-κB p65, MAPK p38, and JNK). One randomized controlled trial showed that patients with hypercholesterolaemia who consumed a purified mixture of anthocyanins (320 mg once a day) for 24 weeks had significantly lower plasma levels of interleukin 1 (IL-1β), C-reactive protein (hsCRP), and soluble vascular cell adhesion molecule-1 (SVCAM-1) than patients receiving a placebo. In addition, the anthocyanin compounds delphinidin 3-O-β-glucoside and cyanidin 3-O-β-glucoside were shown to exhibit additive or synergistic anti-inflammatory effects. They have a dose-dependent inhibitory effect on CRP (C-reactive protein) production induced by IL-6 and IL-1β in the HepG2 cell line and the LPS-induced secretion of VCAM-1 in porcine iliac artery endothelial cell lines [66]. The anti-inflammatory activity of fruits has been confirmed in many in vitro studies, which indicates that anthocyanins inhibit activity of the inflammatory enzyme cyclooxygenase-2 (COX-2), responsible for prostaglandin synthesis. An in vitro study comparing the anti-inflammatory activities of extracts of sour cherries, sweet cherries, and berries found the highest COX-2-inhibiting activity for extracts of sweet cherry (47%), blueberry (46%), strawberry (43%), and raspberry (41%). The inhibition of cyclooxygenase 1 and 2 by anthocyanins has been confirmed to increase as the number of sugar residues in the molecule decreases and is strongest for the free aglycone cyanidin [67]. A similar dependency was observed for the antioxidant activity of anthocyanins [67,68,69,70]. The antioxidant activity of fruits is influenced not only by the content of anthocyanins but by their structure as well, i.e., the degree of hydroxylation and glycosidation. Cyanidin derivatives present in raspberries (mono-, di-, and triglycosides) exhibit the strongest antioxidant properties among all known anthocyanins. Among analyzed anthocyanin compounds, cyanidin triglicoside (3-O-(xylosyl glucosyl)-5-O-galactoside) was observed to display dose-dependent anti-inflammatory activity [71].

Another mechanism of the anti-inflammatory activity of anthocyanins is their effect on mitogen-activated protein kinases (MAPK). These are proteins involved in cell survival and cellular processes, such as proliferation, differentiation, migration, and apoptosis. The mitogen-activated protein kinase (MAPK) pathway is involved in the transmission of signals from the cell membrane to the nucleus in response to a variety of stimuli and in various intracellular processes affecting the growth, differentiation, and stress response of cells. This pathway has also been shown to play a key role in tumor progression [72]. Other researchers suggest that peonidin 3-O-glucoside may inhibit the metastasis of lung cancer cells via a mechanism of impairment of the phosphorylation of extracellular signal-regulated kinase (ERK)1/2, a member of the family of mitogen-activated protein kinases (MAPK) and also inhibits the activator protein-1 (AP-1) [73]. Interesting reports on the activity of pomegranate extract, a rich source of anthocyanin compounds, include the findings of American researchers who showed a positive effect of the extract at a concentration of 20 µg/mL on the UV-B-dependent phosphorylation of the MAPK pathway (ERK l/2, protein p38, and JNK 1/2) in human epithelial keratinocytes [74]. The antioxidant activity of fruit containing anthocyanins has been shown to depend not only on their total content but also on their structure—the degree of hydroxylation and glycosylation. An example is the presence of a hydroxyl group on the B ring of the flavonoid, which modulates the antioxidant activity of compounds [75]. 

One of the fruits most abundant in anthocyanins is black raspberry (*Rubus occidentalis* L.). The activity of black raspberry extract was analyzed in terms of the treatment and prevention of colorectal and oesophageal cancer. To determine the specific mechanisms underlying the potential anti-inflammatory effect of black raspberry extract, primary endothelial cells were used to model endothelium-leukocyte interactions. Black raspberry extract inhibited the TNF-α/IL-1β-induced translocation of NFκB p65; PGE2 production; the regulation of the COX-2, ICAM-1, and VCAM-1 genes; protein expression; and the binding of leukocytes in human oesophageal microvascular endothelial cells [76]. 

Another fruit containing mainly anthocyanins (primarily cyanidin glycosides) is elderberry (*Sambuci fructus*), used in folk medicine as an antipyretic, diuretic, diaphoretic, analgesic, and anti-inflammatory agent [77,78]. The anti-free radical effect of fruit is ascribed to the presence of anthocyanins [79]. The anti-inflammatory effects of the extracts of elderberry fruits have been demonstrated. In animal models, a methanol and n-hexanol elderberry extract had an anti-inflammatory effect comparable to that of diclofenac, inhibiting the carrageenan-induced oedema of rat paws [80]. 

Anthocyanins from sour cherries have been shown to suppress indicators of pain caused by inflammation in rats [81]. 

The anti-inflammatory effect of anthocyanins from chokeberries (*Aronia melanocarpa* L.) involves blocking LPS-induced expression of the proteins iNOS and COX-2 in RAW 264.7 cells, which leads to the inhibition of the release of TNF-α and PGE2 and a significant reduction in NO production [82]. Among the molecular mechanisms responsible for the protective effect of chokeberry extract on ethanol-induced stomach ulcers in a rat model, we can distinguish a decrease in the inflammatory process (the infiltration of inflammatory cells and oedema formation) and a decrease in the levels of MCP-1, MDA, NF-κB and TNF-α [83]. A reduction in the plasma levels of cytokines TNF-α and IL-6, following the administration of chokeberry extract, was also observed in rats with artificially induced hypertension [84]. In a study by Zapolska et al. [85], chokeberry extract exhibited anti-inflammatory activity and inhibited TNF-α-stimulated transcription of the ICAM-1 and VCAM-1 genes, thereby reducing the expression of adhesion molecules in human aortic endothelial cells in an experiment. In addition, the extract inhibited activation of nuclear transcription factor by TNF-α and reduced ROS production in human aortic endothelial cells.

Studies have shown that anthocyanins exert an anti-inflammatory effect by inhibiting the expression of COX-2 in lipopolysaccharide-activated RAW 264 cells or inhibit inducible iNOS and mRNA expression in mouse LPS-activated J774 macrophages. Among five anthocyanidins, delphinidin and cyanidin inhibited the LPS-induced expression of COX-2, in contrast to peonidin and malvidin. Delphinidin, the strongest inhibitor, caused the dose-dependent inhibition of COX-2 expression at both the mRNA level and the protein level [86,87]. In addition, delphinidin was shown to inhibit all three subfamilies of mitogen-activated protein kinase (MAPK): stress-activated c-Jun amino terminal kinase (JNK), extracellular signal-regulated kinase (ERK), and p38 kinase [86].

#### 4.1.6. Flavan-3-ols

**Figure 2:**
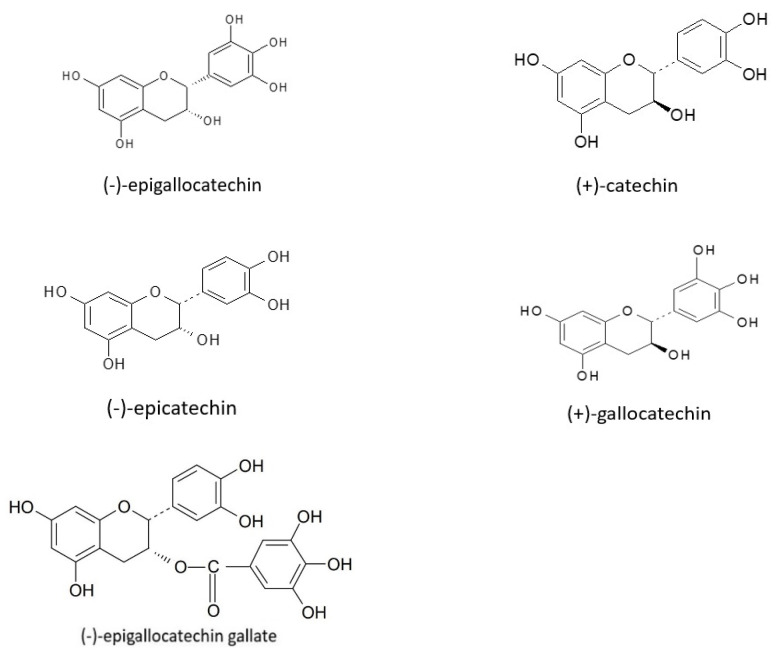
Structural formulas of selected flavan-3-ols.

Flavonoids also include flavan-3-ols, present in many fruits, such as blueberries, strawberries, gooseberries, sour cherries, black grapes, cranberries, and apples [88,89]. According to some authors, catechin and epicatechin are present in higher concentrations in dark grapes, blackberries, apricots, raspberries, and some varieties of apples. Gallocatechin, epigallocatechin, and epigallocatechin gallate have been found in grapes (Figure 2), [90,91]. 

Hanrly et al. [89] showed that the flavan-3-ols present in fruits in the highest concentrations are catechin, epicatechin, epicatechin gallate, gallocatechin, epigallocatechin gallate, and epigallocatechin. The increase in the incidence of diseases with free-radical etiology has led to an intensive search for natural antioxidants, of which many fruits are a rich source. The health-promoting activity of flavan-3-ols/catechins relies mainly on their antioxidant effects. The antioxidant effect of catechin can be effective in preventing and treating cardiovascular and other inflammatory diseases [92,93,94]. In patients with neurodegenerative diseases, the use of flavan-3-ols has been shown to eliminate unfavorable changes in neurons through the inhibition of oxidative stress, the scavenging of reactive oxygen species, and the activation of antioxidant enzymes [95]. Flavan-3-ols have inhibited carcinogenesis induced by inflammation in various models [96]. Flavan-3-ols also exhibit anti-inflammatory effects in human intestinal diseases, influencing cellular signaling pathways associated with oxidative stress, such as nuclear factor kappa B (NF-κB), mitogen-activated protein kinases (MAPK), nuclear factor erythroid 2-related factor 2 (Nrf2), and the signal transducer and activator of transcription 1/3 (STAT1/3) [97]. Dietary supplementation with catechin effectively reduced the risk of inflammation in patients with allergic rhinitis. Catechin reduces expression of TSLP (thymic stromal lymphopoietin) and NF-κBp65 in the nasal mucosa of mice with allergic rhinitis. It inhibits the TSLP expression and activation of the NF-κB signaling pathway in human nasal epithelial cells (HNEpC) [98].

### 4.2. Elagitannins

Another group of polyphenolic compounds in fruits is tannins, present mainly as phenolic polymers. Tannins are able to precipitate proteins and have astringent properties. They have varied molecular weights, and some of them, especially hydrolyzing tannins, are water-soluble. These tannins include esters of ellagic and gallic acid. A second type is condensed tannins present in grapes, known as proanthocyanidins. They are responsible for sensory impressions upon the consumption of fruit, such as an astringent taste [24,25].

**Figure 3:**
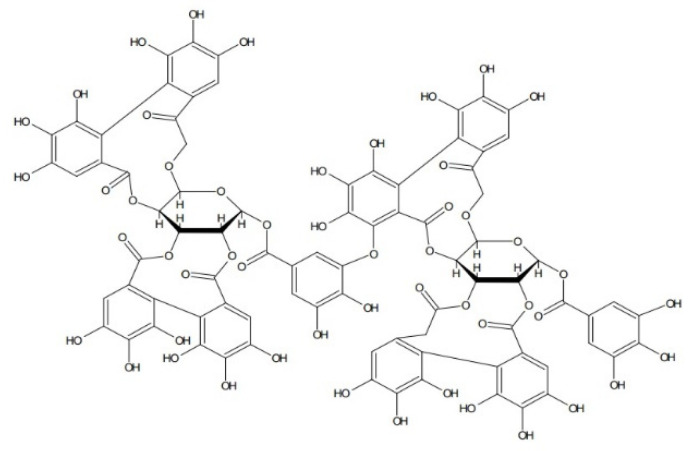
Sanguiin H-6.

Ellagitannins are hydrolyzing tannins, derivatives of ellagic acid. They accompany anthocyanin compounds in most fruits, especially those of the genus Rubus (raspberries, blackberries, and cloudberries) and the genus Fragaria (strawberries), as well as the species Punica granatum (pomegranate). It is significant that there are more ellagitannins in the seeds than in the fruits, e.g., in raspberries [99]. Mullen et al. [100] and Ross et al. [101] showed that the ellagitannin fraction isolated from raspberries, including sanguiin H-6, has stronger antioxidant properties than the anthocyanin fraction (Figure 3). An analysis was conducted of the antioxidant activity of extracts from the fruits of black raspberry (*Rubus occidentalis* L.) with or without crushed seeds, obtained using 60% ethanol or water. The aqueous extract from fruits with crushed seeds had the strongest antioxidant effect, with the lowest IC50 in the DPPH assay (130 μg/mL) [102]. Vuorela et al. [103] tested the antioxidant activity of an extract of raspberry fruits by the DPPH method. For the concentrations of 0.5 mg/mL and 1 mg/mL of extracts, the results were 47 and 56%. A comparison was also made of the activity of the ellagitannin and anthocyanin fractions, which at concentrations of 0.5 mg/mL and 1 mg/mL showed antioxidant activity at levels of 66% and 88% (ellagitannins) and 29 and 48% (anthocyanins). 

Jean-Gilles et al. [104] published the results of a study carried out in arthritic rats that were orally administered an extract of red raspberry fruits (*Rubus idaeus* L.) at 30 and 120 mg/kg BW for 30 days. The higher dose of red raspberry extract was shown to inhibit the inflammatory process and to reduce cartilage damage and bone resorption. The dominant polyphenols in the raspberry extract were ellagitannins. 

The antioxidant and anti-inflammatory activity of a black raspberry seed extract and a grape seed extract were compared. The tannin fraction of the black raspberry seed extract consists of ellagitannins, including sanguiin H-6 and H-10 isomers and galloyl-bis-HHDP glucose isomer, while the grape seed extract contained proanthocyanidins. The antioxidant activity of the tannin fraction of the black raspberry seed extract, evaluated using FRAP (ferric reducing antioxidant power), DPPH (2,2-diphenyl-1-picrylhydrazyl), and ABTS (2,2′-azino-bis(3-ethylbenzothiazoline-6-sulfonic acid) assays, was much higher than in the case of the grape seed extract. The anti-inflammatory effect was analyzed by measuring the ability to inhibit LPS-induced NO production in RAW 264.7 cells. The extracts with higher antioxidant activity (from black raspberry seeds) were also confirmed to have a stronger inhibitory effect on NO than the grape seed extract [105]. 

The anti-inflammatory effect of extracts of pomegranates (*Punica granatum* L.), which are rich in ellagitannins, was analyzed. An extract of pomegranate shell, containing the ellagitannin punicalagin, exhibited anti-inflammatory properties in Caco-2 cells in an in vitro model of the human intestinal epithelium. It acted on the level of transcription of pro-inflammatory genes (encoding IL-6 and MCP-1) as well as protein secretion (IL-6, IL-8 or MCP-1) [106].

### 4.3. Phenolic Acids

**Figure 4:**
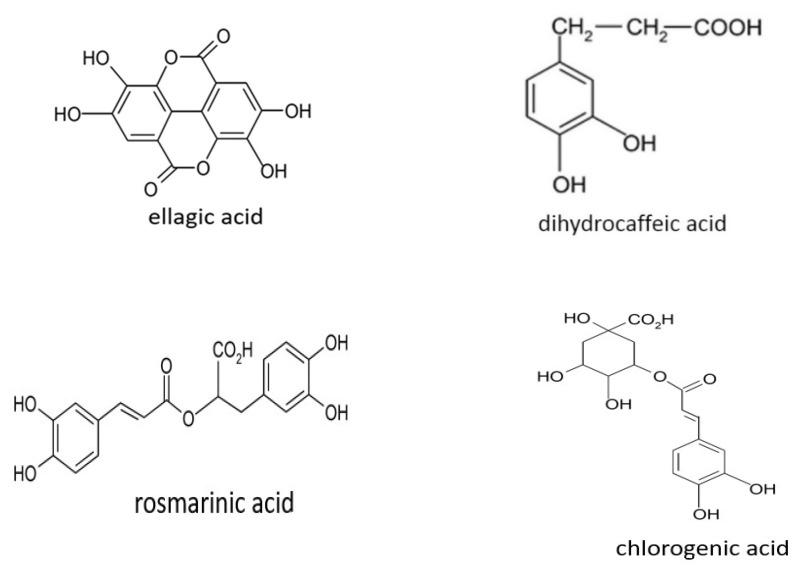
Structural formulas of selected depsides and other phenolic acids.

Another important group of phytocompounds present in fruit is phenolic acids, which account for nearly a third of polyphenols in the diet. They are present in fruits in bound form as esters and glycosides. They can be divided into two main types according to their structure: hydroxyl derivatives of benzoic or cinnamic acid [25]. Most berries, particularly blackberries, raspberries, blueberries, and cranberries, as well as apples, oranges, and sour cherries, are rich in hydroxybenzoic and hydroxycinnamic acid derivatives (Table 3 and Table 4) and their depsides and other phenolic acids (Figure 4).

Phenolic acids play a key role as antioxidants. They can also reduce tissue damage caused by oxidative stress due to chronic illnesses and cancer. Phenolic acid derivatives differ in the methoxylation and hydroxylation patterns of their aromatic rings. They are mainly present in bound form and have strong antioxidant properties due to the reactivity of the phenolic part—the hydroxyl substituent in the aromatic ring. This is a group of compounds capable of scavenging reactive oxygen species, including hydroxyl and superoxide radicals, thereby reducing the number of lipid peroxide radicals and preventing lipid peroxidation. Phenolic acids act as strong antiradical agents owing to their redox properties, which makes them effective hydrogen donors and metal chelators. Some hydroxybenzoic acid derivatives are currently used as additives to reduce the oxidation of nutrients and ensure the high nutritional quality of food products [26]. An early study on the relationship between the structure and activity of phenolic acids and their derivatives showed that hydroxycinnamic acid derivatives have stronger antioxidant properties than benzoic acid derivatives [24]. This potential stems from the existence of a propionic side chain in cinnamic derivatives and a conjugated double bond in their side chains [26].

Phenolic acids, such as caffeic, ferulic, and ellagic acid, are widespread as glycosides in fruits. Ellagic acid is present in high concentrations in fruits of the family *Rosaceae* (blackberries, strawberries, and raspberries) and in cranberries, goji berries, and pomegranates [107]. In raspberries, ellagic acid accounts for 88% of polyphenols determined as the sum of flavonols and phenolic acids [108]. Ellagic acid comprises more than half of the total phenolic content in berry fruits, such as raspberries, and is present in free and compound form as glucosides and ellagitannins esterified with glucose. The inclusion of rich sources of ellagic acid and its derivatives in the diet, such as cranberries, raspberries, blackberries, strawberries, and grapes, has health benefits. The biological effects of ellagic acid include antiviral, anti-inflammatory, antiproliferative, and antioxidant activity [109]. Gallic acid is abundant in bananas, blueberries, lemons, lychee fruit, pears, and apples [110]. It is one of the strongest antioxidants in which three hydroxyl groups are attached to a carbon atom in a benzene ring [111]. 

Interesting reports on the biological activity of Korean black raspberry include the results of a study in which the fraction containing high concentrations of 3,4-dihydroxybenzoic acid exerted the strongest anti-inflammatory effect in vivo in a model of the inhibition of oedema of rat paws [112].

An experiment conducted by Ruifeng et al. [113] found that chlorogenic acid alleviates the symptoms of mastitis in mice through the inhibition of the NF-κB signaling pathway, mediated by toll-like receptor 4 (TLR4). Rowan berries (*Sorbus aucuparia* L) are a source of chlorogenic and neochlorogenic acids [114]. Hydroxycinnamic acid derivatives, especially caffeic, and m-coumaric and p-coumaric acids are present in black currants (*Ribus nigrum* L.) [115]. Another study analyzed the composition of 14 varieties of blueberries grown in China and their anti-inflammatory activity in a model of LPS-induced TNF-α and IL-6 secretion in RAW 264.7 macrophages [116]. Correlation analysis showed that blueberries which had higher concentrations of phenolic acids exhibited stronger antioxidant and anti-inflammatory properties.

Huang et al. [117] confirms that chlorogenic acid can improve the function of human umbilical vein endothelial cells by inhibiting oxidative damage and pro-inflammatory cytokines induced by TNF-α. Chlorogenic acid reduces levels of ROS and xanthine oxidase and increases the concentrations of superoxide dismutase and haem oxygenase-1 in endothelial cells. Chlorogenic acid was additionally shown to induce the TNF-α-stimulated expression of intercellular adhesion molecule-1, vascular cell adhesion molecule-1, and monocyte chemotactic protein 1. Other researchers report that ellagic acid lowers the ROS level in the vascular endothelium [118]. Corbett et al. [119] and Rogerio et al. [120] found that ellagic acid derivatives or extracts from plants containing ellagic acid inhibit carrageenan-induced oedema in rodents. The anti-inflammatory activity of gallic acid was demonstrated in similar research models in rodents, through the mechanisms of scavenging of superoxide anions, the inhibition of the release and the activity of myeloperoxidase, and interference with the formation of active NADPH oxidase [121]. Supplementation with phenolic acids, including gallic, caffeic, ferulic, and protocatechuic acids, in metabolic syndrome induced by a high-fructose diet reduced plasma levels of markers of inflammation in the serum, such as IL-6, IL-8, and TNF-α [122]. Anti-inflammatory effects of caffeic and ellagic acid have also been reported by Chao et al. [123]. The enrichment of the diet with a mixture of phenolic acids reduced the expression of mediators of inflammation, such as IL-6, IL-1-β, and TNF-α. Da Cunha et al. [124] demonstrated the anti-inflammatory properties of caffeic acid and its derivatives as well as the strong inhibition of the LPS-induced expression of nitric oxide synthase (iNOS) in RAW 264.7 macrophages.

### 4.4. Stilbenes

Stilbenes are polyphenols that have gained in importance in recent years. Certain berries and grapes as well as their leaves, stems and roots are particularly rich in stilbenes [125,126]. Stilbenes have been studied extensively in terms of their antioxidant, antibacterial, antifungal, cardioprotective, neuroprotective, anti-ageing, and anti-tumor properties [127,128,129,130,131]. 

**Figure 5:**
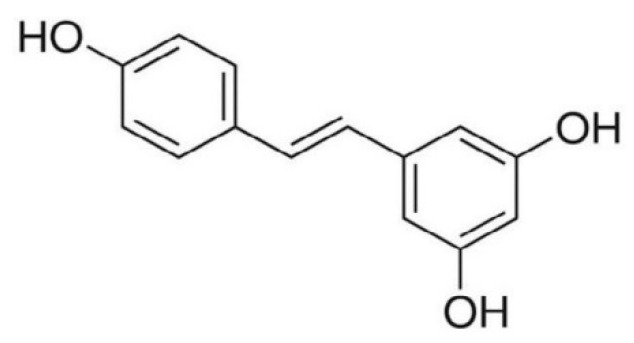
Structural formulas of resveratrol.

The best described compound belonging to this group is resveratrol (Figure 5). It is present in high concentrations in red grape skins and in cranberries, blueberries, and plums. The ‘French paradox’ is directly linked to the resveratrol in red wine. The French have relatively low rates of coronary disease despite the fact that their diet is relatively rich in saturated fats. In addition, the incidence of cardiac infarction in France is about 40% lower than in other European countries. Regular, moderate consumption of grape products, especially red wine, is believed to play a key role in preventing heart disease [25]. 

Resveratrol exhibits anti-inflammatory and antioxidant effects [132]. It can act in synergy with other natural antioxidants, such as vitamins C and A. This natural phytoalexin has the ability to reduce the risk of cardiovascular disease through the regulation of the production of vasodilators and vasoconstrictors, the inhibition of oxidative stress and ROS generation, anti-inflammatory effects, the inhibition of modification of low-density lipoproteins, and antiplatelet activity [97]. Apart from its antioxidant activity, resveratrol induces quinone reductase, a phase II detoxification enzyme [133]. It exerts an anti-inflammatory effect by inhibiting the activity of cyclooxygenase, thereby influencing the arachidonic pathway of prostaglandins, which stimulate tumor cell growth [134]. Resveratrol inhibited LPS-induced pro-inflammatory enzymes and pro-inflammatory cytokines by reducing the phosphorylation of NF-κB, CREB, and MAPK in an mTOR-dependent manner in LPS-stimulated mouse BV-2 microglia cells [135]. Other studies showed that resveratrol reduces the expression of the mediators of inflammation TNF-α, IL-8, and MCP-1 in LPS-stimulated monocytes [136]. Resveratrol promotes the conversion of RNS by limiting the activity of nitric oxide synthase, decreases the cytotoxicity of NO, and reduces the occurrence of inflammation. Its effects also involve blocking the activity of cyclooxygenase and phospholipase A2 [137].

### 4.5. Lignans

Another group of polyphenols, present in low concentrations in fruits, mainly strawberries and cranberries, is lignans. In the digestive tract, lignan molecules are converted to compounds (enterodiol and enterolactone) that exhibit both oestrogenic and anti-oestrogenic properties. Lignans are included among phytoestrogens [138]. They have been shown to exert a synergistic inhibitory effect, together with quercetin and resveratrol in the diet, on oesophageal cancer development in humans. Deoxypodophyllotoxin, a flavolignan, inhibits the LPS-induced expression of iNOS by activating NF-kappa B in RAW 264.7 macrophages [139]. An in vivo study in mice showed that a lignan present in the fruit of magnolia berry (*Schisandra chinensis*), schisandrin B, can eliminate inflammation by reducing the expression of pro-inflammatory cytokines TGF-β1 and TNF-α and the activation of eNOS pathways [140]. The results of another study indicate the role of neolignans obtained from the seeds of C. pinnatifida as potential anti-inflammatory and antioxidant agents inhibiting the secretion of NO and TNF-α in RAW 264.7 cells [141].

## 5. Mechanism of the Anti-Inflammatory Action of Essential Oil Components, Vitamins, Minerals, and Other Compounds in Fruits

### 5.1. Essential Oil Components

Citrus fruits, such as lemons, are a very important dietary component due to their role in preventing obesity, diabetes, cardiovascular disease, and some cancers [142]. The results of a study by Kil-Nam et al. [143] indicate that essential oil obtained from *Citrus medica* L. inhibits LPS-stimulated inflammation by blocking the NF-κB, JNK, and ERK pathways in macrophages, which is evidence of its effectiveness as an anti-inflammatory agent. The main component of the oil, limonene, is the most commonly occurring terpene in nature, present in oranges, mandarins, lemons, limes, and grapefruits. The antioxidant and anti-inflammatory properties of terpenes, which modulate transcription factors, such as NF-κB [144], have been extensively described in the literature. Research conducted by Li et al. [145] demonstrated the anti-inflammatory activity of the peels and pulp of selected varieties of pear, correlated with high content of triterpenes. Terpene compounds present in apple peel reduce the expression of the IP-10 gene associated with the development of inflammation and inflammatory bowel disease [146].

### 5.2. Vitamins

Oranges, grapefruits, sour cherries, apples, and other fruits contain vitamins C, E and A in the form of carotenoids. These vitamins contribute to the normal functioning of the immune system and, in this way, reduce the risk of inflammation in the body. Due to their reducing properties, they are strong antioxidants and help to alleviate the effects of oxidative stress in cardiac disease, diabetes, and certain cancers [69,147]. Berries and black currants are also rich in vitamins C, E, and B, with higher concentrations of vitamins than strawberries, raspberries, and gooseberries. Black currants and strawberries are a particularly rich source of ascorbic acid [69,147,148]. Literature data indicate that the content of vitamin C in black currants (*Ribes nigrum*) can range from 120 to 215 mg/100 g, depending on the variety and on cultivation conditions [11,34,149]. Strawberries and citrus fruits are also very rich sources of vitamin C (80 and 16–60 mg/100 g, respectively) [147,150]. Other fruits, such as apples, pears, and plums, contain low concentrations of vitamin C (3–6 mg/100 g) [151]. The content of vitamin C in fruits depends on multiple factors, including the species, variety, climate conditions, weather, ripeness, region, and storage time and conditions [67,147,148]. The content of vitamin C has been shown to be closely correlated with the antioxidant capacity of citrus fruits [152]. Rosehips are also rich in vitamin C, with higher concentrations than strawberries, oranges, and peaches [151,153]. The stability of ascorbic acid in rosehips has been shown to be higher in the matrix than in extracts, and flavonoids from rosehips may prevent oxidation of ascorbic acid [153]. Vitamin C is a strong antioxidant and free radical scavenger which prevents free-radical damage to DNA, tissues, and cell membranes. A population study of healthy men aged 60–79 demonstrated the anti-inflammatory effect of vitamin C. An elevated plasma concentration of vitamin C due to the consumption of fruit and vegetables and the use of supplements was shown to be inversely correlated with the levels of inflammatory markers, such as C-reactive protein and tissue plasminogen activator antigen [154]. Vitamin C may alleviate the symptoms of upper respiratory diseases, especially the common cold, and reduce their duration [155]. Research to date has shown that increasing the dosage of vitamin C to 200 mg/day does not reduce the frequency of colds in the overall population, but supplementation with vitamin C is justified in people during short-term intense physical exertion, in which it was shown to reduce the frequency of colds by 52% [156]. Vitamin C deficiency has been observed in patients with COVID-19, and, in accordance with the latest reports, supplementation with this vitamin is recommended in patients in high risk groups for death from coronavirus infection [157,158,159]. The mechanism of the antioxidant action of vitamin C stems from its free-radical deactivating properties, so that it prevents DNA damage [160] and inhibits the development of inflammation [161]. Vitamin C increases the production of extracellular collagen, which plays a key role in immune system function [162]. Jang et al. [163] showed that a diet rich in vitamin C can reduce the level of pro-inflammatory cytokines, including TNF-a and IL-6, through the significant down-regulation of hepatic mRNA expression. 

Vitamin E exerts an antioxidant effect by scavenging lipid peroxide radicals in vivo and in vitro and prevents the oxidation of fatty acids contained in the phospholipids of biological membranes and plasma lipoproteins [164]. Muraoka et al. [165] reported that α-tocopherol reduces the hypoxia-induced activation of NF-kB. Most studies focus on the effect of vitamin E on T-cell activity and the modulation of the Th1 response, but vitamin E also influences other immune/inflammatory cells. The use of vitamin E alleviates diseases with concomitant inflammation: viral, bacterial, and allergic diseases as well as asthma [166]. Studies in animals have shown that vitamin E in the form of tocotrienol and α-tocopherol can inhibit the secretion of IL-1 and IL-6 and neutralize free radicals before they are able to activate NF-κ B, thereby inhibiting cytokine production and the expression of COX-2 [167]. In addition, supplementation with vitamin E in people and in animal models has been shown to reduce lipid peroxidation, the release of pro-inflammatory cytokines, and the concentrations of chemokine IL-8 and plasminogen activator inhibitor-1 (PAI-1), as well as reducing the adhesion of monocytes to the endothelium. Moreover, it reduces CRP levels in patients with cardiovascular disease and in patients with risk factors for its development [168].

Carotenoids are pigments present in the diet in fruit. Citrus fruits (oranges and mandarins) and peaches are sources of β-cryptoxanthin and zeaxanthin. Watermelon is a rich source of lycopene, while β-carotene is present in bananas. Large quantities of carotenoids, primarily lutein and β-carotene, are found in black currants, rosehips, chokeberries, and seaberries [169]. Fruits containing carotenoids in smaller amounts include kiwi, lemon, apple, pear, apricot, sour cherries, melon, strawberries, and grapes [170,171]. In addition to antioxidant activity, α-carotene, β-carotene, γ-carotene, lycopene, and β-cryptoxanthin also exhibit anti-inflammatory properties. Immune cells are particularly susceptible to oxidative stress. β-carotene has been shown to protect the immune system against reactive oxygen species [172]. Adequate intake of β-carotene in the diet contributes to a stronger cellular response by increasing the number of monocytes producing MHC II molecules [173]. Inadequate intake of β-carotene has an adverse effect on immune function and increases the incidence of infectious diseases [174]. Vitamin A takes part in immune system development and plays a regulatory role in cellular and humoral immune processes. Retinoic acid plays a key role in the regulation of the differentiation, maturation, and function of cells of the innate immune system. Vitamin A has been shown to be essential to the development and differentiation of colonic CD169+ macrophages [175]. Vitamin A deficiency increases susceptibility to infection, possibly by decreasing the level of cathepsin G in the azurophilic granules of neutrophils. Twining et al. [176] showed lower activity of two cathepsin G-like enzymes (28 and 24 kDa) in rats with a vitamin A-deficient diet compared to neutrophils of rats with a complete diet of unlimited vitamin A. Diet supplementation with retinoids increases the release of tissue stores of latent TGF-β 1, which is essential in the wound healing process [177]. Semba et al. [178] presented research results demonstrating that children with vitamin A deficiency show reversible immune abnormalities in T-cell subgroups. Decreased absorption of vitamin A in the intestines and the release of retinol from the liver are observed during inflammation, which can limit the availability of the vitamin in the tissues. During infections, vitamin A can be lost in large quantities in the urine. Low plasma levels of retinol (hyporetinolaemia) have been detected in n children and adults with infectious diseases, e.g., measles, malaria, diarrhea, and HIV [179].

### 5.3. Minerals

Fruits, such as strawberries, apples, blueberries, sour cherries, and grapes, are rich in micro- and macroelements. The key minerals present in these fruits are potassium, magnesium, calcium, phosphorus, iron, sodium, copper, zinc, aluminum, selenium, cadmium, and manganese. The highest concentrations of phosphorus, calcium, sodium, and iron are found in berries. Some microelements, such as iron, selenium, zinc, copper, and manganese, act as cofactors for antioxidant enzymes and take part in redox processes in the body, reducing the ROS level in cells [147,180]. Other studies have shown high content of potassium, calcium, and magnesium but small amounts of sodium, in fruits of the genera *Ribes* and *Rubus*, and the highest concentration of phosphorus in currants [181]. The consumption of fruits such as bananas can help to meet requirements for potassium, magnesium, copper, and boron [182]. Many studies have shown an inverse relationship between magnesium concentrations in the diet and the occurrence of inflammation [183,184,185]. Sources of iron in the diet include dates, black currants, avocado, cranberries, gooseberries, and citrus fruits [186,187]. Iron performs an important function in the immune response. Studies in animals have shown that iron deficiency causes inflammation and a stronger inflammatory response to LPS in comparison with mice with normal iron levels [188,189]. Zinc, copper, and selenium play an important role in maintaining redox homeostasis, which is essential to immune function, and changes in their levels can lead to oxidative stress and the development of inflammation [190,191,192].

### 5.4. Fiber

Dietary fiber is a crucial component of a healthy diet. Observational studies support associations between dietary fiber intake and inflammation. Long-term dietary fiber intake was inversely associated with plasma levels of IL-6 and TNF-α-R2 (TNF-α-receptor-2), in post-menopausal women [193] and decreased risk of inflammation and mortality in kidney disease [194]. Anti-inflammatory activity of a fiber rich diet is a potential effect of the modification of pH and the permeability of the gut and reduction in inflammatory compounds production [195]. Pectin is a major fruit prebiotic that has been extensively studied and shown to promote an anti-inflammatory colonic microbiota ecosystem. Chung et al. [196] tested bacterial strains for their ability to utilize apple pectin and confirmed that *E. eligens* strongly promoted the production of the anti-inflammatory cytokine IL-10 in vitro. Gut microbiota composition associated with anti-inflammatory effects differs between individuals and is dependent on a variety of factors like diet and genetics [197]

### 5.5. Other Compounds

Research on the fruit of the Cornelian cherry (*Cornus mas* L.) of the family *Cornaceae* indicates that the main fractions responsible for its anti-inflammatory effect are iridoids. The secoiridoid cornuside isolated from the Cornelian cherry inhibited the LPS-induced production of NO, PGE2, TNF-α, IL-6, and IL-1β; reduced the mRNA expression of COX-2 and iNOS; and inhibited the translocation of NF-κBp in vitro [198]. Glycosides isolated from *Cornus mas* were found to inhibit oedema and the secretion of IL-1, IL-6, and TNF-α in peritoneal macrophages and PGE2 in the plasma of rats [199]. An aqueous extract of the fruit inhibited the LPS-induced expression of COX-2 and iNOS in RAW 264.7 macrophages. It also inhibited PGE2 synthesis and NO production and reduced the level of NF-κB in the cell nucleus [200]. 

Research has shown that anti-inflammatory compounds also include polysaccharides from the fruit of Chaenomeles speciosa, which inhibits the gene induction of TNF-α or IFN-γ in LPS-stimulated RAW 264.7 macrophages [201].

Ursolic acid is a pentacyclic triterpenoid, which is widespread in the skins of fruits, including apples. Some studies indicate that ursolic acid has anti-inflammatory effects in chronic kidney disease and kidney fibrosis. Ursolic acid has been shown to decrease the occurrence of inflammation and inhibit the expression of inflammatory cytokines (TGF-β, IL-6, and TNFα) in the muscle cells of mice with chronic kidney disease [202,203].

The anti-inflammatory effect of the accessory fruits of dog rose (*Rosa canina* L.) is due to galactolipid [204]. The presence of vitamins and flavonoids supports the action of galactolipid [205]. Clinical trials on the anti-inflammatory properties of rosehips have been conducted for several years. In patients with chronic arthritis and rheumatoid arthritis taking powdered rosehips, improvement in the condition of the joints and pain relief have been observed [206,207,208].

## 6. Conclusions

Inflammation plays an important role in the pathogenesis and course of many diseases, including cardiovascular diseases, neurodegenerative diseases, musculoskeletal diseases, cancers, diabetes, and allergies. Active compounds present in fruit can play an important role in health maintenance by reducing the risk of inflammation and resolving it. Components of fruits can modulate/suppress the process at several stages. Further research is needed on the anti-inflammatory and antioxidant mechanisms of the action of fruit, particularly their active components. Additional clinical trials are necessary to determine their potential in preventing and treating inflammation, which will make it possible to develop strategies for treatment and supporting treatment.

## Figures and Tables

**Table 1:** Structural formulas of selected flavonoid aglycons and glycosides.

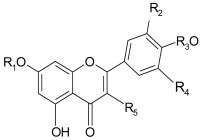					
Compound	R_1_	R_2_	R_3_	R_4_	R_5_
Kaemferol	H	H	H	H	OH
Kaemferol 3-O-glucoside	H	H	H	H	O-glucose
Kaemferol 7-O-glucoside	glucose	H	H	H	OH
Kaemferol 7-O-rhamnoside	rhamnose	H	H	H	OH
Quercetin	H	OH	H	H	OH
Quercetin 3-O-glucoside	H	OH	H	H	O-glucose
Quercetin 3-O-galactoside	H	OH	H	H	O-galactose
Quercetin 3-O-rhamnoside	H	OH	H	H	O-rhamnose
Quercetin 7-O-glucoside	glucose	OH	H	H	OH
Rutin	H	OH	H	H	O-rutinose
Quercetin 3-O-glucuronide	H	OH	H	H	O-glucuronic acid
Apigenin	H	H	H	H	H
Luteolin	H	OH	H	H	H
Myricetin	H	OH	H	OH	OH

**Table 2:** Structural formulas of selected anthocyanins.

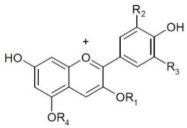				
Compound	R_1_	R_2_	R_3_	R_4_
Cyanidin	H	OH	H	H
Cyjanidin 3-O-glucoside	glucose	OH	H	H
Cyjanidin 3-O-rutinoside	Glucose + rhamnose	OH	H	H
Cyjanidin 3-O-(2^G^-glukosylorutinoside)	glucose + rhamnoza + glucose	OH	H	H
Cyjanidin 3-O-soforoside	glucose + glucose	OH	H	H
Cyjanidin 3-O-sambubioside		OH	H	H
Cyjanidin 3,5-O-diglucoside	glucose	OH	H	glucose
Pelargonidin 3-O-glucoside	glucose	H	H	H
Pelargonidin 3-O-rutinoside	glucose + rhamnose	H	H	H
Pelargonidin 3,5-O-diglucoside	glucose	H	H	glucose
Malvidin 3-O-glucoside	glucose	OCH_3_	OCH_3_	H
Delfinidin 3-O-glucoside	glucose	OH	OH	H

**Table 3:** Structural formulas of selected benzoic acid derivatives.

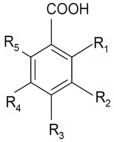					
Compound	R_1_	R_2_	R_3_	R_4_	R_5_
benzoic acid	H	H	H	H	H
salicylic acid	OH	H	H	H	H
m-hydroxybenzoic acid	H	OH	H	H	H
p-hydroxybenzoic acid	H	H	OH	H	H
2,3-dihydroxybenzoic acid	OH	OH	H	H	H
β-rezorcylic acid	OH	H	OH	H	H
gentysinic acid	OH	H	H	OH	H
γ-rezorcylic acid	OH	H	H	H	OH
protocatechic acid	H	OH	OH	H	H
α-rezorcylic acid	H	OH	H	OH	H
gallic acid	H	OH	OH	OH	H
2,4-dimetoxybenzoic acid	OCH_3_	H	OCH_3_	H	H
veratric acid	H	OCH_3_	OCH_3_	H	H
vanillic acid	H	OCH_3_	OH	H	H
syryngic acid	H	OCH_3_	OH	OCH_3_	H

**Table 4:** Structural formulas of selected cinamonic acid derivatives.

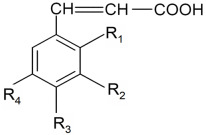				
Compound	R_1_	R_2_	R_3_	R_4_
cinamonic acid	H	H	H	H
p-cumaric acid	H	H	OH	H
m-cumaric acid	OH	H	H	H
o-cumaric acid	H	OH	H	H
ferulic acid	H	OCH_3_	OH	H
isoferulic acid	H	OH	OCH_3_	H
caffeic acid	H	OH	OH	H
synapic acid	H	OCH_3_	OH	OCH_3_
4-metoxycynamonic acid	H	H	OCH_3_	H
3,4-dimetoxycynamonic acid	H	OCH_3_	OCH_3_	H
2,4-dimetoxycynamonic acid	OCH_3_	H	OCH_3_	H
